# Refined Approach to the Treatment of Caseous Calcification of the Mitral Annulus: A Case Report

**DOI:** 10.7759/cureus.76337

**Published:** 2024-12-24

**Authors:** Maria Beyer, Rishabh Kasarla, Seth Shoap, Erik Beyer

**Affiliations:** 1 Cardiothoracic Surgery, University of Texas Health Science Center, San Antonio, USA; 2 College of Medicine, Nova Southeastern University Dr. Kiran C. Patel College of Allopathic Medicine, Fort Lauderdale, USA; 3 Orthopaedic Surgery, Penn State Health Milton S. Hershey Medical Center, Hershey, USA; 4 Cardiothoracic Surgery, Palmetto General Hospital, Hialeah, USA

**Keywords:** bovine pericardial patch, caseous calcification of the mitral annulus (ccma), embolization, mitral regurgitation (mr), mitral valve prolapse (mvp), mitral valve replacement, partial debridement, stroke symptoms, systemic embolization, thrombogenesis

## Abstract

Caseous calcification of the mitral annulus (CCMA) is a rare variant of mitral annular calcification (MAC), in which the core of the calcification undergoes a caseous transformation. CCMA can cause dysfunction of the mitral valve or embolization of caseous material, requiring surgery. There is currently no clear consensus on the optimal treatment strategy for CCMA. Attempts to address the condition, including surgical removal of calcification or mitral valve replacement, have faced certain challenges. This case details a refined approach consisting of partial debridement of the calcification and subsequent annular reconstruction with a bovine pericardial patch prior to surgical valve replacement.

## Introduction

This article was originally presented as a Case Video at The American Association for Thoracic Surgery's Mitral Conclave Workshop in Boston on May 14, 2022. 

The mitral valve annulus serves as the anatomical junction between the left atrium and left ventricle, anchoring the mitral valve leaflets. Mitral annular calcification (MAC) is a chronic condition characterized by calcium deposition within the fibrous tissue of the mitral annulus [1]. Caseous calcification of the mitral annulus (CCMA) is a rare variant of MAC, involving degenerative, calcified, and necrotic changes within the cardiac valve skeleton [2].

The rarity of CCMA highlights the need for greater awareness among clinicians, as its uncommon occurrence can lead to under-recognition, misdiagnosis, and challenges in management. Its echocardiographic prevalence is approximately 0.64% among patients with MAC and 0.068% in the general population [3]. CCMA is more common in older individuals, women, and patients with hypertension, chronic kidney disease, or altered calcium-phosphate metabolism [4]. The pathophysiology of CCMA is heterogeneous, often involving calcific material that may be densely compacted, friable, or caseous. The extent of involvement varies, potentially affecting the mitral leaflets, chordae, papillary muscles, or adjacent cardiac structures [4].

Diagnosing CCMA presents notable challenges, as it is frequently misinterpreted as an abscess, tumor, or infective vegetation on the mitral valve [5]. CCMA lacks a specific clinical presentation, making multimodal imaging essential for diagnosis, often as part of the evaluation for an intracardiac mass [5]. Modalities such as computed tomography (CT) are valuable for their capacity to detect the calcified envelope [6]. On CT imaging, CCMA typically presents as a well-defined, oval, or crescent-shaped hyperdense mass with peripheral calcification, predominantly located along the posterior mitral annulus [5].

Despite its often asymptomatic nature, CCMA poses significant risks, including embolization, conduction abnormalities, and mitral valve dysfunction [5,7,8]. The embolization of caseous material, independent of atrial fibrillation, is a frequent complication, contributing to cardioembolic strokes that lead to severe neurological deficits [7]. The proposed mechanisms of embolization include dislodgement of small calcified fragments, surface ulceration leading to thrombus formation and subsequent embolization, or fistulization of caseous necrosis into the lumen of the left atrium or left ventricle [5]. Additionally, CCMA’s proximity to the atrioventricular node and conduction system can result in bradyarrhythmias and advanced atrioventricular blocks [5].

Surgical intervention for CCMA is complex due to its anatomical location near critical cardiac structures, risk of embolization, and variable extent of calcification. While conservative management is sufficient in most cases such as with asymptomatic patients, surgery is warranted for cases with significant valvular dysfunction, systemic embolization, or when it is impossible to rule out the possibility of a tumor [7,8]. The decision to perform mitral valve replacement rather than repair may depend on the extent of calcification and the presence of symptoms, which reflect the severity and complexity of the condition [5]. Adding to its complexity, CCMA has the potential to spontaneously resolve and revert to MAC but may also recur even after surgical excision [5].

Given its potential to cause severe mitral dysfunction and systemic complications, CCMA presents a critical area of study. This report aims to expand current knowledge by detailing a severe presentation of CCMA involving mitral regurgitation (MR) and systemic embolization. The case highlights a refined surgical approach to address the challenges of the risk of embolization and injuring close-by cardiac structures and emphasizes the importance of tailored interventional strategies for this rare and complex condition. 

## Case presentation

**Figure 1:**
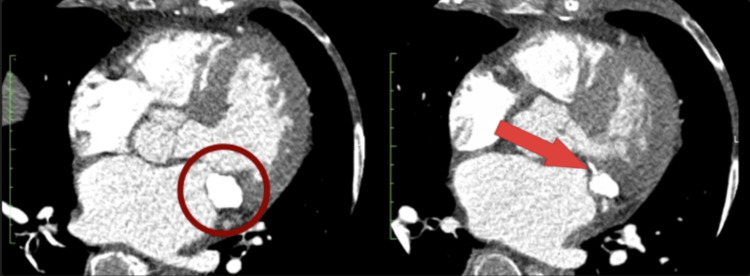
Imaging with CT revealed a large, calcified vegetation on the posterior aspect of the mitral annulus. Also shown is its proximity to the left circumflex artery CT: computed tomography

An 85-year-old female with a history of hypertension and obesity presented with signs of a stroke, which resolved after treatment with tissue plasminogen activator (tPA). An echocardiogram revealed a flail leaflet on the posterior aspect of the mitral valve, diffuse leaflet thickening, and significant MR (Video 1). As a part of the stroke workup, imaging with a CT scan was performed. It revealed a large (2.13 cm by 1.42 cm) calcified vegetation with a soft core on the posterior aspect of the mitral annulus, which was identified as a CCMA (Figure 1). The presentation of a stroke, severe MR from a ruptured chord, and mitral valve prolapse (MVP) due to the involvement of the CCMA on the posterior leaflet of the mitral valve indicated the need for surgical intervention. 

**Video 1:** Echocardiogram reveals a flail posterior mitral valve leaflet with diffuse thickening and associated MR MR: mitral regurgitation

**Figure 2:**
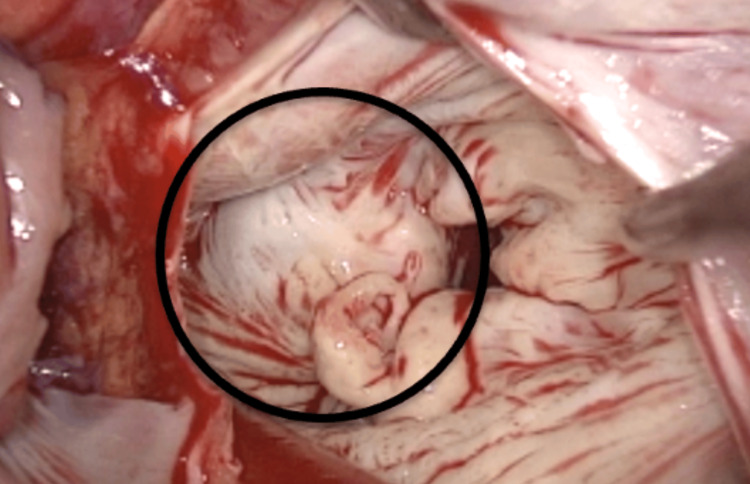
The calcified vegetation on the posterior aspect of the mitral annulus identified as the CCMA CCMA: caseous calcification of the mitral annulus

During the surgical intervention, the CCMA was identified along the posterior aspect of the mitral annulus (Figure 2), consistent with its location observed on the pre-operative CT scans. The valve was completely excised, and the calcification, characterized by a soft, "toothpaste-like" core, was debrided precisely enough to restore the native size of the mitral annulus while minimizing the risk of atrioventricular groove separation or damage to the circumflex coronary artery. The remaining exposed CCMA and the debrided area of the mitral annulus were covered with a double layer of bovine pericardium and secured circumferentially. The bovine tissue also served as a sturdy platform on which to seat the prosthetic mitral valve.

**Figure 3:**
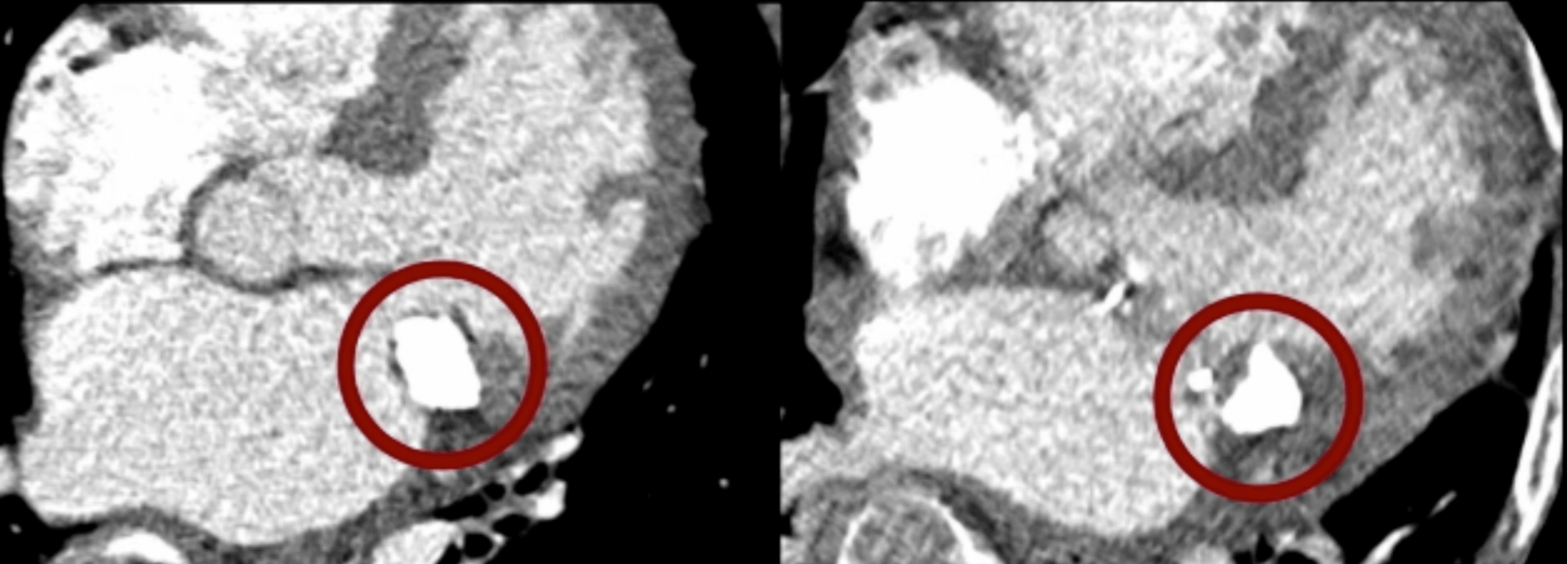
A comparison of pre-operative (left) and post-operative (right) CT scans shows a reduction in the size of the mass following partial resection CT: computed tomography

The surgery was successful in removing a significant portion of the CCMA, replacing the mitral valve, and resolving the patient's associated symptoms (Video 2). There were no indications of further embolization of the caseous material. Follow-up CT scan reveals a partially resected mass, reduced in size compared to its pre-operative dimensions (Figure 3). The patient had an adequate post-operative course and was discharged 12 days following surgery. At one month's follow-up, she had excellent functional status. She has had no cardiovascular complications following surgery and appears well three years post-operation.

**Video 2:** The surgical treatment involved partial debridement of the CCMA, which was covered with a double-layer bovine pericardial patch, providing a foundation for the placement of the prosthetic mitral valve CCMA: caseous calcification of the mitral annulus

## Discussion

In our case, the decision was made to proceed with surgical intervention rather than conservative management. Typically, conservative management is sufficient, as CCMA is most often an incidental finding and asymptomatic [9]. Although there is no consensus on the optimal approach, current evidence supports conservative treatment when the diagnosis is clear and there is no obstruction to left atrial emptying [10]. 

Indications for surgical intervention include significant mitral valve dysfunction, embolic events, or the inability to exclude the possibility of a tumor [5]. In this case, our patient presented with severe mitral regurgitation and a cerebrovascular accident, necessitating surgical excision. Despite successful surgical management, CCMA requires long-term follow-up due to the risk of recurrence [5].

When surgical intervention is required, a critical decision must be made between mitral valve repair and replacement. Mitral valve replacement offers a definitive solution to the dysfunction caused by CCMA. In this case, mitral valve replacement was necessary due to the ruptured chordae, the large size of the mass, the severity of calcification, and the extent of valvular damage.

The available literature provides limited guidance on whether minimal or total debridement is the optimal surgical approach for CCMA [11]. Below, we summarize key considerations and challenges associated with prevailing surgical interventions. 

Mitral valve replacement with minimal or no debridement reduces the risk of myocardial injury compared to extensive calcium removal. However, this approach leaves the caseous calcification exposed, which may continue to pose a risk of embolization. Furthermore, securing a prosthetic valve to calcified tissue is technically challenging and increases the likelihood of post-operative complications, such as paravalvular leaks [12]. 

In contrast, extensive debridement or complete calcium removal aims to eliminate embolic material but is associated with significant risks [6]. These include potential injury to the circumflex coronary artery, myocardial damage that may lead to atrioventricular groove separation, and cerebral embolism in the immediate postoperative period [11-13]. Balancing these risks underscores the complexity of surgical decision-making in CCMA management. 

Our goal was to balance the risks associated with minimal and excessive debridement, specifically minimizing the potential for embolism and post-operative paravalvular leak linked to minimal debridement, while avoiding myocardial damage and anatomical injury associated with excessive debridement. To achieve this, we adopted a refined approach that involved debriding the calcification just enough to restore the native size of the mitral annulus. We then reconstructed the annulus by covering the remaining calcification with a bovine pericardial patch. This patch effectively sealed the exposed CCMA to prevent further embolization and provided a stable platform for securing the prosthetic mitral valve.

## Conclusions

In conclusion, partial debridement of the calcification and subsequent annular reconstruction with a bovine pericardial patch prior to surgical valve replacement offers a refined approach that preserves existing anatomy and prioritizes patient safety. This tailored strategy balances the risks of extensive versus minimal debridement, offering a viable alternative for managing complex cases of CCMA. While the approach requires specialized surgical expertise, which may limit its generalizability, it highlights the importance of individualized surgical planning in achieving optimal outcomes. This report adds to the growing body of knowledge on interventional strategies for CCMA, highlighting the need for further research to evaluate long-term outcomes, foster the development of innovative and standardized approaches, and promote multicenter studies to validate this strategy and explore its potential inclusion in future guidelines. This strategy worked well for our patient, leading to a full recovery with resolution of mitral valve dysfunction and no evidence of further embolization.
